# Editorial: Role of matrix metalloproteinases and other inflammatory mediators in the disruption of the intestinal tight junction barrier

**DOI:** 10.3389/fimmu.2023.1194827

**Published:** 2023-04-26

**Authors:** Rana Al-Sadi, Raz Abdulqadir, Thomas Y. Ma

**Affiliations:** Department of Medicine, Division of Gastroenterology and Hepatology, Penn State Milton S. Hershey Medical Center, Hershey, PA, United States

**Keywords:** matrix metalloproteinases (MMP), intestinal tight junction (TJ) barrier, inflammatory bowel disease, barrier function dysregulation, inflammatory mediator, bile acids

The intestinal epithelium is a single cell layer within the gut lumen that serves an important protective function. It acts as a barrier to prevent harmful luminal contents and antigens from crossing into the intestinal tissue. Defective intestinal tight junction barrier (TJ), manifested by increased intestinal permeability, has been shown to contribute to the pathogenesis of inflammatory bowel disease (IBD) including Crohn’s disease (CD) and ulcerative colitis (UC). Matrix metalloproteinases (MMPs) have been implicated in the pathogenesis of intestinal inflammation in IBD and other inflammatory conditions of the gut. Dysregulated expression of MMPs and other pro-inflammatory cytokines, including TNF-α, IL-1β, IL-6, IFN-γ, have been shown to have a disruptive effect on the intestinal TJ barrier function. Enhancement or maintenance of the intestinal TJ barrier function has been shown in several clinical studies to be a potential therapeutic approach in preventing intestinal inflammation.

This Research Topic, “*Role of Matrix Metalloproteinases and Other Inflammatory Mediators in the Disruption of the Intestinal Tight Junction*”, invited Original Research, Reviews, Mini Reviews, and Opinion articles that address MMPs and/or pro-inflammatory cytokines regulation of intestinal TJ barrier function and IBD pathogenesis, and studies investigating interventions, solutions, or therapeutic targeting to prevent the inflammatory response of MMPs and other cytokines. There were 3 articles contributed by 18 authors addressing insights into understanding of the effects of different inflammatory mediators, especially MMPs, with a particular focus on the inflammatory bowel disease (IBD) and gut barrier dysfunction.

MMP-7, also called matrillysin-1, is the smallest MMP with proteolytic activity against a broad range of substrates, including pro-TNFα and Galectin-3. MMP-7 expression has been shown to be increased in the inflamed colonic mucosa of IBD. However, it is not known if MMP-7 affects gut epithelial TJ proteins in IBD. Xiao et al. showed that MMP-7 is highly expressed in the colonic biopsies of patients with UC. In this study, the mRNA and protein expression of MMP-7 in the colon were found to be markedly elevated in 6 experimental colitis models examined: dextran sulfate sodium (DSS) rats, trinitrobenzene sulfonic acid (TNBS) rats, DSS mice, TNBS mice, IL10-/- mice, and adoptive T-cell transfer mice. The expression of MMP-7 contributed to the stimulation of pro-inflammatory cytokines, including TNFα, IL-1β, IL-4, IL-13, and LPS, in immune and epithelial cells in the colon. They also suggested that MMP-7 disruption of intestinal epithelial TJ barrier function was mediated by downregulation of claudin-7, but not claudin-1, -8, or -15 protein expression. Animal studies in MMP-7 deficient mice suggested that MMP-7 mediates epithelial barrier dysfunction, and that MMP-7 deficiency ameliorates colitis by targeting claudin-7. Furthermore, MMP-7 monoclonal antibody administration preserved the intestinal barrier function and claudin-7 expression and ameliorated inflammation in DSS-treated mice and TNBS-treated rats. These studies suggested MMP-7 as an important regulator of chronic gut inflammation and a potential therapeutic target for the treatment of IBD.

An extensitive review from Opdenakker et al. addressed the recent literature related to MMPs in IBD since 2016. This review contained far-reaching information and discussion about the benefits and limitations of animal models for IBD research, and provided updates of diagnostic procedures and mechanistic insights into current therapies, including clinical trials with antibodies against MMP-9. The review suggest that the MMP-9 may have an effect rather than a cause in IBD pathogenesis. MMP-9 is postulated to cleave substrates at the level of intestinal barrier (denatured collagens, claudins, occludins, precursor defensins, actins, cadherins, the cytokines TNF and VEGF and the chemokine ligand CXCL-8/IL-8 and cellular receptors) and may contribute mechanistically to tissue damage. The authors also explained some of the reasons why MMP-9 inhibition studies were stopped in clinical studies. Although promising data were published, the clinical studies of Andecaliximab (a chimeric recombinant IgG4 against MMP-9, formerly named GS-5745, Gilead Sciences) was stopped in IBD for futility reasons. The authors referred to their own studies showing that neutralizing antibodies against MMP-9 had immunological effects that might be detrimental in clinical settings of endotoxinemia, and concluded that these animal model studies did not provide evidence for a beneficial role of MMP-9 inhibition for IBD. This review also referred to the mechanisms of MMP-9 regulation and its therapeutic applications *in vitro* and *in vivo*. For example, in the acute phase of IBD, p38 kinase and NF-κB have been shown to mediate the MMP-9 induced disruption of intestinal epithelial TJ barrier, while in the chronic phase of IBD, the toll-like receptor (TLR) activation of N-κB pathway was not essential for intestinal fibrosis development. The authors also reviewed recent developments about new therapeutics targeting MMPs during chronic phases of IBD.

The bile acids (BA) have been implicat to play important physiological and pathophysiological roles in IBD. Calzadilla et al. summarized the current knowledge about the roles for bile acids as inflammatory mediators and how they contribute to IBD by altering the immune responses and epithelial barrier function in the intestine. This review addressed BA receptors and their mechanisms of action that were shown to affect immune response related to IBD, including G-protein bile acid receptor 1, sphingosine 1 phosphate receptor 2, Vitamin D Receptor among others. The authors also reviewd the changes in conjugated and unconjugated bile acids levels in serum and feces in IBD patients. There have been several studies examining the effects of bile acids on intestinal epithelial permeability, apoptotic signaling, and cytokine secretion. Most of the studies reviewd in this article pointed to unconjugated BA as having negative or no effect on intestinal barrier function in both *in vitro* studies and *in vivo* models of intestinal inflammation. On the other hand, most taurine conjugated BA studies showed beneficial effects towards intestinal inflammation. Current literature on BA as therapeutics in human IBD is limited, but several studies in preclinical animal models have suggested that some BAs improve barrier function, decrease apoptotic signaling, and an overall decrease in disease severity. However, the mechanisms by which BAs contribute to the pathophysiology or severity of intestinal inflammation remain unknown.

The articles published in this Research Topic enhances our understanding of inflammatory mediators in modulating gut barrier dysfunction in the context of IBD. The authors are sincerely acknowledged for their outstanding contributions to making this collection a great success.

## Author contributions

RA-S prepared the draft. RA and TM contributed to editing. RA-S and TM approved submission.

